# Knowledge of HIV prevention biomedical tools among African immigrants in France: the Makasi project

**DOI:** 10.1093/eurpub/ckac130.173

**Published:** 2022-10-25

**Authors:** K Coulibaly, A Gosselin, S Carillon, A Ravalihasy, M Bousmah, C Taeron, R Mbiribindi, A Desgrées du Loû

**Affiliations:** 1 Ceped, Université Paris Cité, IRD, Inserm, France; 2 CNRS, French Collaborative Institute on Migrations, Aubervilliers, France; 3 Ined, National Institute for Demographic Studies, Aubervilliers, France; 4 Arcat, Paris, France; 5 Afrique Avenir, Paris, France

## Abstract

**Background:**

In France, post-exposure prophylaxis (PEP) and pre-exposure prophylaxis (PrEP) have been available for several years. However, there is still no evidence on the level of knowledge of these HIV prevention tools among immigrants from sub-Saharan Africa living in precarious situations, a population particularly affected by HIV. The aim of this study is to describe the knowledge of these tools in this population and analyse the factors associated with this knowledge.

**Methods:**

The data mobilized are from the Makasi interventional research that was conducted between 2018 and 2020 among immigrants from sub-Saharan Africa in precarious situations in the greater Paris area. Using data collected from 601 participants, we described levels of knowledge of HIV treatment effectiveness, treatment as prevention (TasP), post-exposure prophylaxis (PEP), and pre-exposure prophylaxis (PrEP), by sex with a chi2 test. We investigated factors associated with their knowledge with logistic regressions adjusted for sociodemographic characteristics, living conditions and sexual behaviors (p ≤ 0.2).

**Results:**

The population surveyed was predominantly men (76%), from West Africa (61%) and in a precarious situation: 69% were unemployed, 74% were undocumented, 46% had no health coverage and 13% were homeless. In this population, knowledge of antiretroviral treatments for HIV prevention was heterogeneous: the effectiveness of HIV treatment was well known (84%), but only half of the respondents (46%) were aware of TasP and very few knew about PEP and PrEP: 6% and 5% respectively. Multivariable-adjusted models showed that these tools was better known by educated people, those who had a social network in France, those who have had access to the health system and those who were exposed to sexual risks.

**Conclusions:**

While sub-Saharan African immigrants know the effectiveness of HIV treatment and use certain prevention tools such as HIV testing, they are not aware of PEP and PrEP.

**Key messages:**

- PEP and PrEP are two HIV prevention tools that are not well known by sub-Saharan African immigrants.
- There is an urgent need to disseminate information about these prevention tools to immigrants.

